# ﻿Key to the North American tribes and genera of herb, rose, bramble, and inquiline gall wasps (Hymenoptera, Cynipoidea, Cynipidae*sensu lato*)

**DOI:** 10.3897/zookeys.1196.118460

**Published:** 2024-03-25

**Authors:** Louis F. Nastasi, Matthew L. Buffington, Charles K. Davis, Andrew R. Deans

**Affiliations:** 1 Frost Entomological Museum, Department of Entomology, The Pennsylvania State University, 501 Agricultural Science & Industries Building, University Park, PA, 16802, USA The Pennsylvania State University University Park United States of America; 2 Systematic Entomology Laboratory, USDA-ARS, c/o National Museum of Natural History, Smithsonian Institution, PO Box 37012, MRC 168, Washington, DC, 20013, USA Systematic Entomology Laboratory, USDA-ARS, c/o National Museum of Natural History, Smithsonian Institution Washington United States of America

**Keywords:** Cecidology, identification, taxonomy

## Abstract

Robust keys exist for the family-level groups of Cynipoidea. However, for most regions of the world, keys to genera are not available. To address this gap as it applies to North America, a fully illustrated key is provided to facilitate identification of the tribes and genera of rose gall, herb gall, and inquiline gall wasps known from the region. For each taxon covered, a preliminary diagnosis and an updated overview of taxonomy, biology, distribution, and natural history are provided.

## ﻿Introduction

Gall wasps (Hymenoptera: Cynipidae*sensu lato*) comprise a fascinating group of gall inducers and inquilines that are associated with a tremendous diversity of host plants, including at least eight families (Ronquist et al. 2015; Azmaz and Katılmış 2020; Buffington et al. 2020). However, the taxonomy of these insects is poorly resolved, and few resources exist to enable their identification. Some recent keys (e.g., Buffington et al. 2020) stand as milestones within systematics of Cynipoidea, as they are heavily illustrated and feature images that highlight important diagnostic characters, but these products only address family-level taxa. Within the gall wasps in particular, no comparable keys exist for genera, and virtually no useful diagnostic tools exist for the North American fauna specifically. The last generic key broadly covering North American cynipids is that of Weld (1952), privately published more than 70 years ago. As a result, a new key is necessary to enable tribal and generic identification of North American cynipids.

Part and parcel to this dilemma is a general observation that the current limits of many cynipid genera themselves are in flux, leaving a difficult situation for providing effective keys. Towards this end, we have addressed this challenge by focusing herein on inquilinous cynipids and those inducing galls on herbaceous and rosaceous plants, therein leaving the oak galling cynipids (tribe Cynipini) for future projects.

Recent revisionary works (e.g., Lobato-Vila and Pujade-Villar 2021) have made the North American cynipid fauna more approachable. Additionally, the cynipid fauna of the United States, Canada, and Mexico exclusive of Cynipini was recently cataloged by Nastasi and Deans (2021). An overview of North American cynipid taxonomy as treated therein is provided in Table 1.

**Table 1.** Overview of North American gall wasp fauna. Species numbers refer to those known from North America; taxonomy, species numbers, and biological data are based on Nastasi and Deans (2021) except for Cynipini, which is derived from Melika et al. (2021). * = raised to subfamily Diplolepidinae in the family Diplolepididae by Hearn et al. (2023).

Taxon	Biology	Nr of spp.
**Tribe Aulacideini**	**Gall inducers on Asteraceae; Lamiaceae**	**21**
Genus *Antistrophus* Walsh	Gall inducers on *Chrysothamnus*, *Lygodesmia*, *Microseris*, *Silphium* (Asteraceae)	10
Genus *Aulacidea* Ashmead	Gall inducers on *Hieracium*, *Lactuca*, *Nabalus*, *Pilosella*, *Rhaponticum* (Asteraceae)	10
Genus *Liposthenes* Förster	Gall inducers on *Glechoma* (Lamiaceae)	1
**Tribe Ceroptresini**	**Inquilines of Cynipini**	**19**
Genus *Buffingtonella* Lobato-Vila & Pujade-Villar	Unknown; presumed inquilines of Cynipini as in *Ceroptres*	1
Genus *Ceroptres* Hartig	Inquilines of Cynipini	18
**Tribe Cynipini**	**Gall inducers on Fagaceae, especially *Quercus***	~ **680**
**Tribe Diastrophini**	**Gall inducers on Rosaceae or inquilines of *Diastrophus* or Diplolepidini**	**25**
Genus *Diastrophus* Hartig	Gall inducers on *Fragaria*, *Potentilla*, *Rubus* (Rosaceae)	14
Genus *Periclistus* Förster	Inquilines in galls induced by *Diplolepis*	7
Genus *Synophromorpha* Ashmead	Inquilines in galls induced by *Diastrophus*	4
Tribe Diplolepidini*	**Gall inducers on *Rosa***	**34**
Genus *Diplolepis* Geoffroy	Gall inducers on *Rosa*	34
**Tribe Phanacidini**	**Gall inducers on Asteraceae**	2
Genus *Phanacis* Förster	Gall inducers on *Hypochaeris*, *Taraxacum* (Asteraceae)	2
**Tribe Synergini**	**Inquilines of Cynipini**	**69**
Genus *Saphonecrus* Dalla Torre & Kieffer	Inquilines of Cynipini	2
Genus *Synergus* Hartig	Inquilines of Cynipini	67

## ﻿Materials and methods

Our taxonomic framework follows Nastasi and Deans (2021); nomenclatural, biological, and distributional data are provided therein for each species in the genera treated in the present work. The skeleton of this key was based on Buffington et al. (2020). Other characters in the present work follow Ronquist et al. (2015) or have been developed through the authors’ taxonomic work on North American Cynipoidea.

For those unfamiliar with cynipoid morphology, we recommend consulting the line drawings of ‘Hymenoptera of the World’ (Goulet and Huber 1993) and Melika (2006). Those more advanced in their knowledge may opt to reference the Hymenoptera Anatomy Ontology (Yoder et al. 2010) or the Phenotype and Trait Ontology (PATO curators 2023), the former of which serves as the primary foundation of the morphological terminology applied herein (Table 2).

**Table 2.** Overview of morphological terminology employed in the key to genera. URLs link to entries in the Hymenoptera Anatomy Ontology (Yoder et al. 2010) or the Phenotype and Trait Ontology (PATO curators 2023).

Term	URL or definition
Areolet	http://purl.obolibrary.org/obo/HAO_0000147
Carina (plural carinae)	http://purl.obolibrary.org/obo/HAO_0000188
Coriaceous sculpture	http://purl.obolibrary.org/obo/HAO_0002379
Eye	http://purl.obolibrary.org/obo/HAO_0000217
Facial radiating striae	http://purl.obolibrary.org/obo/HAO_0001770
Fore wing	http://purl.obolibrary.org/obo/HAO_0000351
Frons	http://purl.obolibrary.org/obo/HAO_0001044
Granular sculpture	http://purl.obolibrary.org/obo/PATO_0001759
Hypopygium	http://purl.obolibrary.org/obo/HAO_0000410
Mesopleural impression	http://purl.obolibrary.org/obo/HAO_0001952
Mesopleuron	http://purl.obolibrary.org/obo/HAO_0000566
Mesoscutum	http://purl.obolibrary.org/obo/HAO_0000575
Metasoma	http://purl.obolibrary.org/obo/HAO_0000626
Metasomal tergite 1	http://purl.obolibrary.org/obo/HAO_0000053
Metasomal tergite 2	http://purl.obolibrary.org/obo/HAO_0000056
Metasomal tergite 3	http://purl.obolibrary.org/obo/HAO_0000057
Metatarsal claw	http://purl.obolibrary.org/obo/HAO_0001927
Notaulus (plural notauli)	http://purl.obolibrary.org/obo/HAO_0000647
Pronotal plate	http://purl.obolibrary.org/obo/HAO_0000838
Pronotum	http://purl.obolibrary.org/obo/HAO_0000853
Punctate-setigenous sculpture	The sculpture that consists of punctation in which each puncture contains a single seta.
Reticulate sculpture	The sculpture that is superficially net-like, consisting of a network of carinae or indentations enclosing polygonal cellules.
Sculpture	http://purl.obolibrary.org/obo/HAO_0000913
Scutellar fovea (plural scutellar foveae)	http://purl.obolibrary.org/obo/HAO_0000916
Seta (plural setae)	http://purl.obolibrary.org/obo/HAO_0002299
Striate sculpture	http://purl.obolibrary.org/obo/PATO_0001410
Suture	http://purl.obolibrary.org/obo/HAO_0000982
Syntergite	http://purl.obolibrary.org/obo/HAO_0000987
Torulus (plural toruli)	http://purl.obolibrary.org/obo/HAO_0000908
Wing cell	http://purl.obolibrary.org/obo/HAO_0001091

Each character is illustrated by color micrographs of museum specimens, which enables stronger recognition of relevant morphology. Images were captured using a Macroscopic Solutions ‘microkit’ (Tolland, CT) imaging station, stacked using Zerene Stacker LLC (Richland, WA), and edited using Adobe Photoshop and/or Adobe Illustrator (San Jose, CA).

Specimens referenced during the production of this key, including those photographed to produce figures, are housed in the Frost Entomological Museum (**PSUC**; University Park, PA) or the United States National Museum of Natural History (**USNM**; Washington, DC). Unique specimen identifiers in the form of catalog numbers (**USNMENT** or **PSUC_FEM** numbers with corresponding barcodes) link each image to specimens housed at the corresponding collection.

## ﻿Results

### ﻿Key to the tribes and genera of herb, rose, bramble, and inquiline gall wasps of North America (Hymenoptera: Cynipoidea)

To verify the applicability of this key to a given specimen, first run unknown individuals through the superfamily key in Goulet and Huber (1993) to confirm the specimen belongs to Cynipoidea, then Buffington et al. (2020) to confirm placement in Cynipidae. This process is critical in that a few North American Figitidae can superficially resemble Cynipidae. We recommend the use of good lighting, diffused through mylar, when using the key; this is especially essential for viewing patterns of cuticular sculpture and characters involving the pronotal plate.

Some North American genera are problematic with regard to their taxonomic status or their true occurrence in the region. Where applicable, these taxa are present in the key or otherwise mentioned in the systematic treatment below. Additionally, many undescribed taxa within the scope of these keys are known, and many taxonomic acts are necessary to stabilize the fauna covered here. Future iterations of the key in this work will address updated taxonomy as it is published, but the present key has been written to be as compatible as possible with all upcoming taxonomic changes known to the authors. We provide provisional taxon diagnoses in the below taxon treatments to facilitate identification of the tribes and genera as they are currently defined; these diagnoses are based only on North American members of each taxon. We expect these diagnoses to change as taxonomic work on the North American cynipid fauna progresses.

**Table:** 

1	Pronotum distinctly short dorsomedially, forming a narrow strip behind head, with medial height (Figs 1–4, pmh) approximately 1/7 or less the lateral height (Figs 3, 4, plh). Pronotal submedial pits absent (Figs 1, 2). Gall inducers on *Rosa* (Rosaceae) or several genera of Fagaceae, especially *Quercus*	**2**
–	Pronotum taller and broader dorsomedially, with medial height (Figs 5, 6, pmh) usually approximately 1/3 the lateral height (Figs 7, 8, plh). Pronotal submedial pits usually present and well-impressed (mep, Fig. 6). Gall inducers on other plants or inquilines in galls	**3**
2	Mesopleuron medially with broad, crenulate transverse impression (Fig. 9, mci). Female hypopygium always distinctly plowshare-shaped (Fig. 10, hyp). Scutellar foveae faint or absent (Fig. 11, scf). Fore wing vein 2r usually with distinct median vein stump projecting distally (Fig. 12, 2r). Gall inducers on *Rosa*	**Diplolepididae: *Diplolepis* Geoffroy**
–	Mesopleuron usually without broad crenulate impression (Fig. 13). Female hypopygium usually not plowshare-shaped (Fig. 14, hyp); if plowshare-shaped (only in *Protobalandricus* Melika, Nicholls & Stone, 2018), then mesopleuron entirely smooth. Scutellar foveae usually distinct (Fig. 15, scf). Fore wing vein 2r usually without distinct stump (Fig. 16, 2r). Gall inducers on Fagaceae, especially *Quercus*	**Cynipini (not keyed further; see taxonomic treatment below)**
3	Metasomal tergites 2 and 3 partially or completely fused into a syntergite, resulting in a metasoma composed of one or two segments (Figs 17, 18, arrows indicate length of syntergite). Inquilines in galls on *Quercus* or female inquilines in galls on Rosaceae	**4**
–	Metasomal postpetiolar terga free and articulated, not forming syntergite and with no single segment especially enlarged (Figs 19, 20, arrows indicate length of longest tergite). Gall inducers on Rosaceae, Asteraceae, or Lamiaceae, or male inquilines in galls on Rosaceae	**8**
4	Metasomal tergites 2 and 3 entirely fused into syntergite (Fig. 22, T2+T3). Head and mesosoma generally roughly sculptured (Fig. 23). Pronotal plate incomplete, at most weakly defined dorsally, and with marginal sutures never reaching anterior margin of mesoscutum (Fig. 21, ppt). Inquilines in galls on *Quercus*	**Synergini: *Synergus* Hartig**
–	Metasomal tergites 2 and 3 often delineated by a distinct suture, with tergite 2 much smaller than tergite 3 and appearing ligulate (Fig. 25), although occasionally entirely fused into syntergite. Body usually less roughly sculptured, often smooth and/or shining (Fig. 26). Pronotal plate complete, well-defined both dorsally and ventrally, and with marginal sutures reaching anterior margin of mesoscutum (Fig. 24, ppt.). Inquilines in galls on *Quercus* or female inquilines in galls on Rosaceae	**5**
5	Metasomal tergites 2 and 3 delineated by a distinct suture, with tergite 2 much smaller than tergite 3 and appearing ligulate (Fig. 27). Mesoscutum with or without abundant setigenous punctation. Female or male inquilines in galls on *Quercus*	**6 (Ceroptresini)**
–	Metasomal tergites 2 and 3 entirely fused into syntergite, at most with a slight indication of a suture delimiting tergite 2 but never with tergites fully separated (Fig. 28). Mesoscutum with distinct setigenous punctation at least anteriorly (Fig. 29). Female inquilines in galls on Rosaceae	**7 (Diastrophini, in part)**
6	Area between toruli depressed and often pubescent (Fig. 30, dep). Metasomal tergite 1 mostly concealed, smooth (Fig. 31, T1). Frons with distinct facial carinae ventral to toruli apparent at least as short ridges below toruli (Fig. 30, fac). Frequently collected	***Ceroptres* Hartig**
–	Area between toruli not depressed and not strongly pubescent (Fig. 32). Metasomal tergite 1 relatively large and ring-like, not concealed, and longitudinally striate (Fig. 33). Frons entirely without facial carinae ventral to toruli (Fig. 32). Very rarely collected	***Buffingtonella* Lobato-Vila & Pujade-Villar**
7	Fore wing with marginal cell closed, with a distinct, complete vein along anterior wing margin (Fig. 34, mcl). Notauli incomplete, absent anteriorly, well developed posteriorly, not apparently widened posteriorly (Fig. 35). Mesoscutum (Fig. 35) coriaceous and punctate-setigenous throughout, and more densely pubescent. Inquilines in galls on *Rosa*	***Periclistus* Förster (females)**
–	Fore wing with marginal cell open, without distinct vein along anterior wing margin (Fig. 36, mcl). Notauli usually complete, always distinctly widened posteriorly relative to anterior width (Fig. 37). Mesoscutum (Fig. 37) smooth to granulate and with fewer setigenous punctures, and less densely pubescent. Inquilines in galls on *Rubus*	***Synophromorpha* Ashmead (females)**
8	Pronotal plate complete, well-defined both dorsally and ventrally, and with marginal sutures distinctly reaching anterior margin of mesoscutum (Fig. 38, ppt). Metatarsal claws with distinct basal lobe (Fig. 39, mtl). Gall inducers or male inquilines in galls on Rosaceae	**9 (Diastrophini, in part)**
–	Pronotal plate usually poorly defined dorsally, never with marginal sutures clearly reaching anterior margin of mesoscutum (Fig. 40, ppt). Metatarsal claws simple and without distinct basal lobe (Fig. 41). Gall inducers on Asteraceae or Lamiaceae	**11**
9	Fore wing with marginal cell closed, with a distinct, complete vein along anterior wing margin (Fig. 42, mcl). Notauli weakly developed, never distinctly complete, and not distinctly widened posteriorly (Fig. 43, not). Mesoscutum more or less densely pubescent throughout (Fig. 43). Male inquilines in galls on *Rosa*	***Periclistus* Förster (males)**
–	Fore wing with marginal cell open, without distinct vein along anterior wing margin (Fig. 44, mcl). Notauli well-developed, usually distinctly complete, and always distinctly widened posteriorly relative to anterior width (Fig. 45, not). Mesoscutum much less pubescent (Fig. 45). Female or male gall inducers on Rosaceae or male inquilines in galls on *Rubus*	**10**
10	Mesoscutum mostly to entirely coriaceous and with distinct setigenous punctures, especially medially (Fig. 46). Male inquilines of *Diastrophus* galls on *Rubus* (females with metasomal tergites 2 and 3 fused into a syntergite, causing metasoma to appear mostly as one large segment)	***Synophromorpha* Ashmead (males)**
–	Mesoscutum mostly smooth and shining, at most weakly coriaceous, and without abundant strong setigenous punctures (Fig. 47). Female or male gall inducers on Rosaceae (females with metasomal tergites 2 and 3 free and articulated and without syntergite)	***Diastrophus* Hartig**
11	Pronotum with submedial pits reduced, usually apparent as a continuous linear depression (Fig. 48). Fore wing with marginal cell partially open, with vein R1 reaching anterior margin of fore wing and continuing along wing margin but not meeting vein Rs (Fig. 49, arrow indicates end of vein R1). Gall inducers on *Taraxacumofficinale* or *Hypochaerisradicata* (Asteraceae)	**Phanacidini: *Phanacis* Förster**
–	Pronotum with submedial pits distinct and well-defined (Fig. 50, mep). Fore wing with marginal cell either entirely open (Fig. 51, arrow indicates end of vein R1 and dotted line indicates margin of fore wing along marginal cell), with vein R1 clearly not reaching wing margin, or entirely closed, with vein Rs reaching wing margin and distinctly reaching vein Rs to enclose cell. Gall inducers on several genera of Asteraceae, or *Glechomahederacea* (Lamiaceae)	**12 (Aulacideini)**
12	Mesopleuron with sculpture primarily or entirely reticulate (Fig. 52, msp), often with fine striae intermediate to rows of reticulate cells (Fig. 53, msp). Second metasomal tergite without distinct patch of setae, at most with a few scattered setae (Fig. 52, 53). Marginal cell of fore wing always open (Fig. 54, mcl). Gall inducers on *Chrysothamnus*, *Lygodesmia*, *Microseris*, or *Silphium* (Asteraceae)	***Antistrophus* Walsh**
–	Mesopleuron with sculpture primarily or entirely transversely striate (Fig. 55, msp). Second metasomal tergite usually with distinct anterolateral patch of pale setae (Fig. 56, T2p). Marginal cell of fore wing usually closed (Fig. 57, mcl), open only in *Liposthenes* Förster. Gall inducers on several genera of Asteraceae or on *Glechomahederacea* (Lamiaceae)	**13**
13	Fore wing with marginal cell closed and usually with areolet distinct (Fig. 58, mcl and aro). Mesoscutum pubescent throughout, always with abundant, closely-set setae and often appearing densely silky (Fig. 59). Gall inducers primarily on Cichorieae (Asteraceae), especially *Lactuca* L	***Aulacidea* Ashmead**
–	Fore wing with marginal cell open and areolet indistinct (Fig. 60). Mesoscutum mostly bare, at most with a few scattered setae (Fig. 61). Gall inducers on *Glechomahederacea* (Lamiaceae)	***Liposthenes* Förster**

**Figures 1–8.**
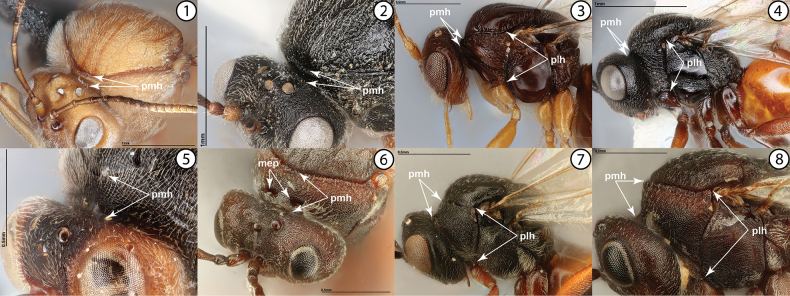
**1***Andricusquercuscalifornicus*, anterodorsal view (USNMENT01231839) **2***Diplolepisbicolor*, anterodorsal view (USNMENT01231831) **3***Dryocosmuskuriphilus*, lateral view (USNMENT01231861) **4***Diplolepisbicolor*, lateral view (USNMENT01231831) **5***Synergusatripennis*, anterodorsal view (USNMENT01231845) **6***Antistrophuslaciniatus*, anterodorsal view (USNMENT01448496) **7***Phanacis* sp., lateral view (USNMENT01448498) **8***Antistrophuslaciniatus*, lateral view (USNMENT01448496). Abbreviations: mep = pronotal submedial pits, plh = pronotum lateral height, pmh = pronotum medial height.

**Figures 9–16.**
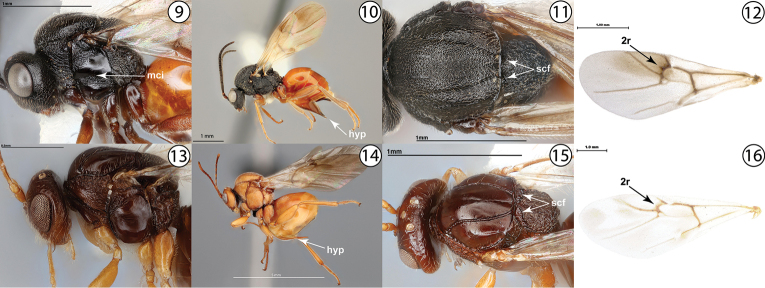
**9***Diplolepisbicolor*, lateral view (USNMENT01231831) **10***Diplolepisbicolor*, lateral view (USNMENT01231831) **11***Diplolepisbicolor*, dorsal view (USNMENT01231831) **12***Diplolepisrosae*, fore wing (USNMENT00655959) **13***Dryocosmuskuriphilus*, lateral view (USNMENT01231861) **14***Andricusquercuscalifornicus*, lateral view (USNMENT01231839) **15***Dryocosmuskuriphilus*, dorsolateral view (USNMENT01231861) **16***Andricuscornigerus*, fore wing (USNMENT00655954). Abbreviations: hyp = hypopygium, mci = mesopleural crenulate impression, scf = scutellar foveae.

**Figures 17–20.**
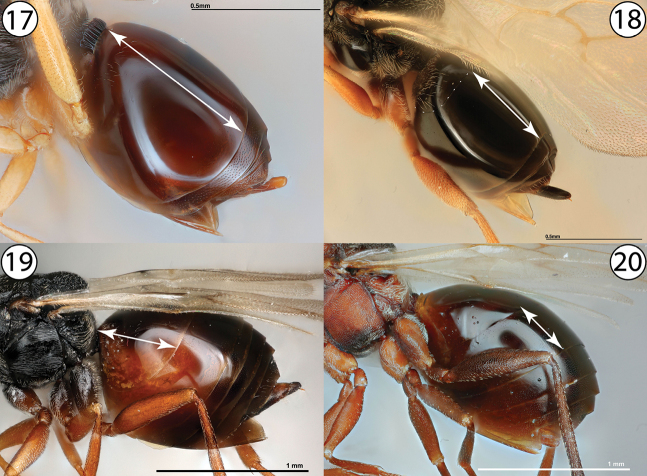
**17***Synergus* sp., metasoma, dorsolateral view (USNMENT01231858) **18***Ceroptres* sp., metasoma, dorsolateral view (USNMENT00917016) **19**Aulacideacf.hieracii, metasoma, lateral view (PSUC_FEM 000253105) **20***Antistrophuspisum*, metasoma, lateral view (PSUC_FEM 000247264). Arrows indicate length of longest metasomal tergite.

**Figures 21–26.**
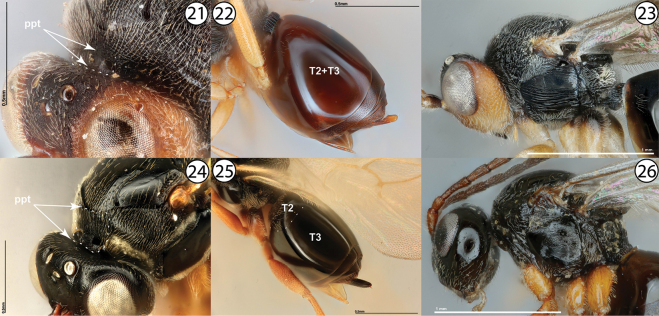
**21***Synergusatripennis*, dorsolateral view (USNMENT01231845) **22***Synergus* sp., metasoma, lateral view (USNMENT01231858) **23***Synergus* sp., lateral view (PSUC_FEM 000079457) **24***Synophromorpha* sp., dorsolateral view (USNMENT01448499) **25***Ceroptres* sp., metasoma, dorsolateral view (USNMENT00917016) **26***Diastrophuskincaidii*, lateral view (PSUC_FEM 000251280). Abbreviations: ppt = pronotal plate, T2 = second metasomal tergite, T2+3 = completely fused second and third metasomal tergites, T3 = third metasomal tergite.

**Figures 27–29.**
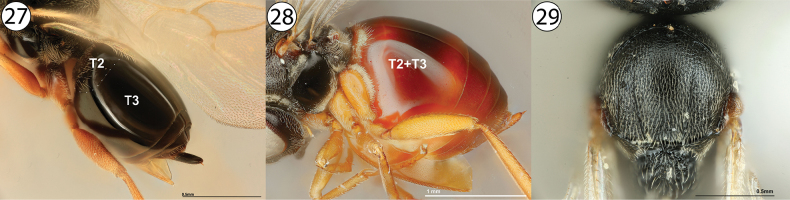
**27***Ceroptres* sp., metasoma, dorsolateral view (USNMENT00917016) **28***Diastrophuskincaidii*, metasoma, lateral view (PSUC_FEM 000251280) **29***Periclistus* sp., lateral view (PSUC_FEM 000250920). Abbreviations: T2 = second metasomal tergite, T2+3 = completely fused second and third metasomal tergites.

**Figures 30–33.**
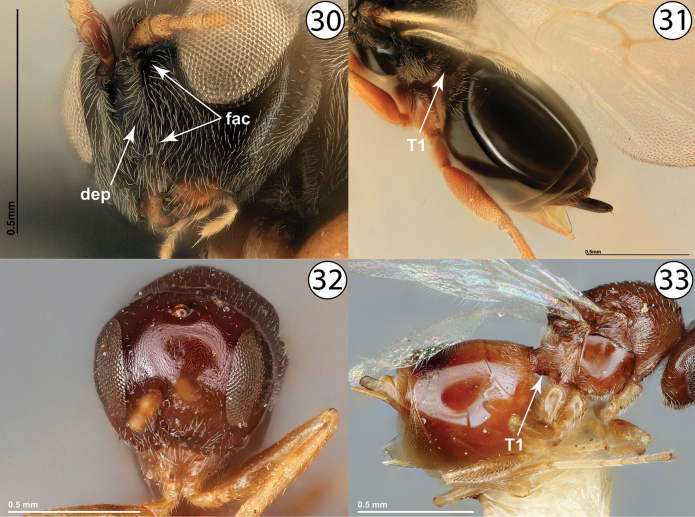
**30***Ceroptres* sp., head, anterior view (USNMENT00917016) **31***Ceroptres* sp., metasoma, dorsolateral view (USNMENT00917016) **32***Buffingtonellapolita*, head, anterior view (USNMENT00892509) **33***Buffingtonellapolita*, lateral view (USNMENT00892509). Abbreviations: dep = depressed intratorular area, fac = facial carinae, T1 = first metasomal tergite.

**Figures 34–37.**
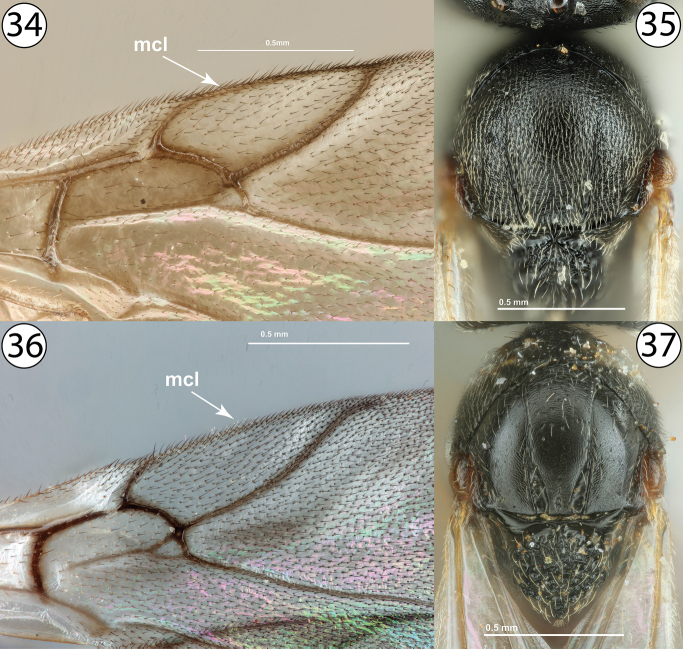
**34***Synergus* sp., fore wing (PSUC_FEM 000079457) **35***Periclistus* sp., dorsal view (PSUC_FEM 000250920) **36***Synophromorpha* sp., fore wing (PSUC_FEM 000250918 **37***Synophromorpha* sp., dorsal view (PSUC_FEM 000250918). Abbreviations: mcl = marginal cell.

**Figures 38–41.**
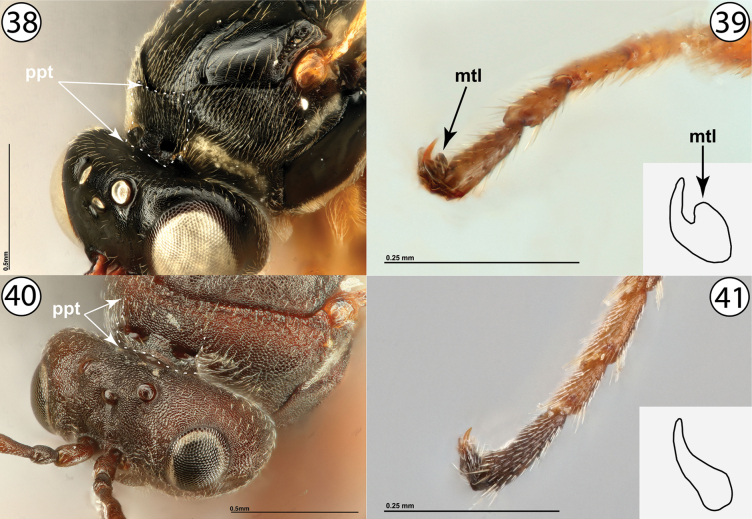
**38***Synophromorpha* sp., dorsolateral view (USNMENT01448499) **39***Diastrophuskincaidii*, tarsal claw (PSUC_FEM 000251280) **40***Antistrophuslaciniatus*, anterodorsal view (USNMENT01448496) **41***Antistrophussilphii*, tarsal claw (CYNANT0048). Abbreviations: mtl = metatarsal claw lobe, ppt = pronotal plate.

**Figures 42–45.**
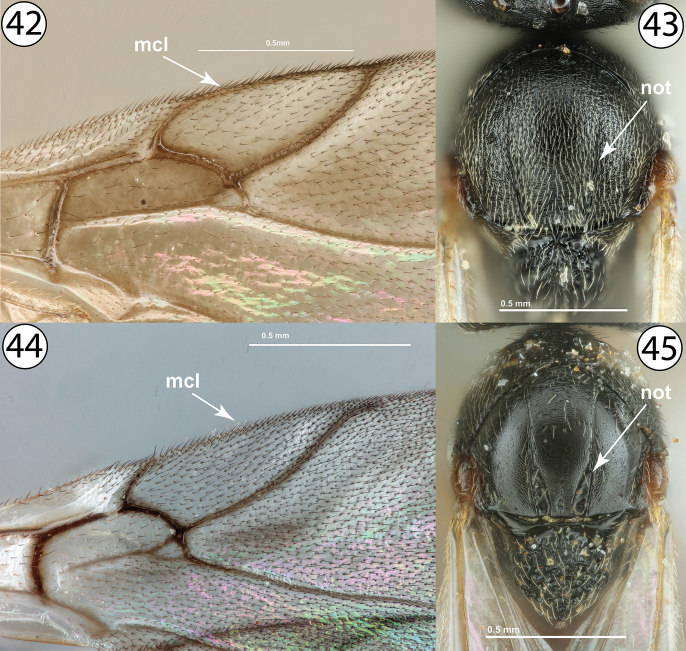
**42***Synergus* sp., fore wing (PSUC_FEM 000079457) **43***Periclistus* sp., dorsal view (PSUC_FEM 000250920) **44***Synophromorpha* sp., fore wing (PSUC_FEM 000250918) **45***Synophromorpha* sp., dorsal view (PSUC_FEM 000250918). Abbreviations: mcl = marginal cell, not = notauli.

**Figures 46, 47.**
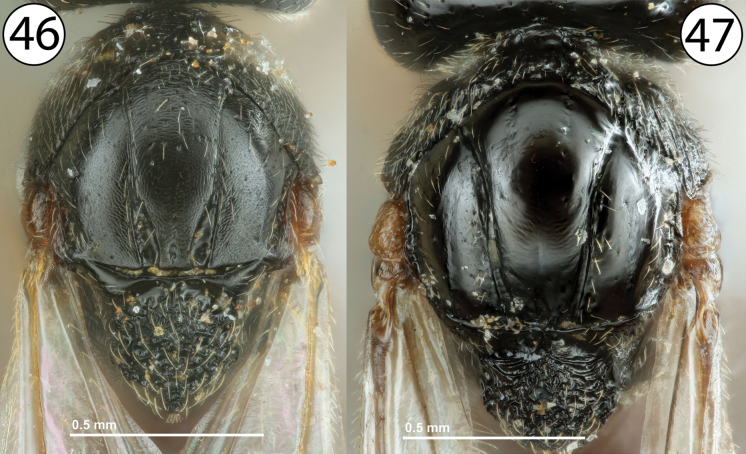
**46***Synophromorpha* sp., dorsal view (PSUC_FEM 000250918) **47***Diastrophuskincaidii*, dorsal view (PSUC_FEM 000251280).

**Figures 48–51.**
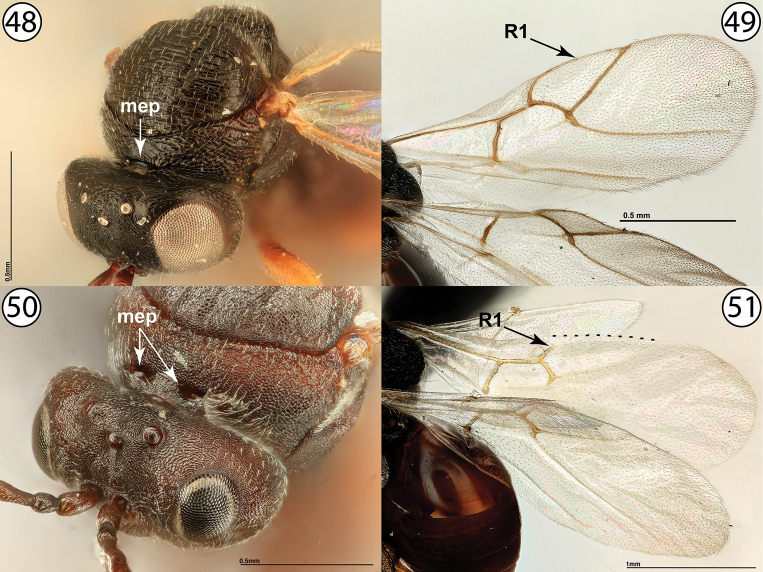
**48***Phanacis* sp., anterodorsal view (USNMENT01448498) **49***Phanacis* sp., wings (USNMENT01231855) **50***Antistrophuslaciniatus*, anterodorsal view (USNMENT01448496) **51***Antistrophuslaciniatus*, wings (USNMENT01448496); dotted line indicates margin of fore wing. Abbreviations: mep = pronotal submedial pits.

**Figures 52–57.**
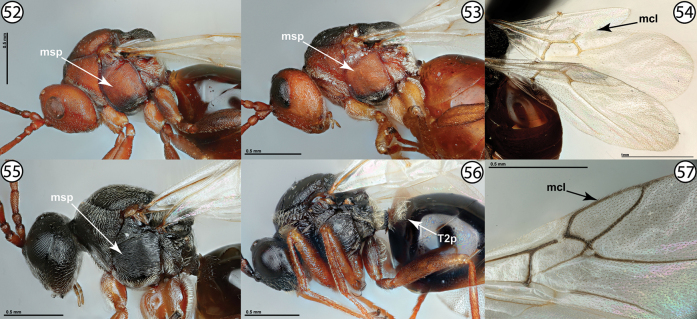
**52***Antistrophuspisum*, lateral view (PSUC_FEM 000247286) **53***Antistrophusmeganae*, lateral view (PSUC_FEM 000248165) **54***Antistrophuslaciniatus*, wings (USNMENT01448496) **55***Aulacidea* sp., lateral view (PSUC_FEM 000247286) **56***Liposthenesglechomae*, lateral view (PSUC_FEM 000248152) **57***Aulacidea* sp., wings (PSUC_FEM 000247286). Abbreviations: mcl = marginal cell, msp = mesopleuron, T2p = setose patch on second metasomal tergite.

**Figures 58–61.**
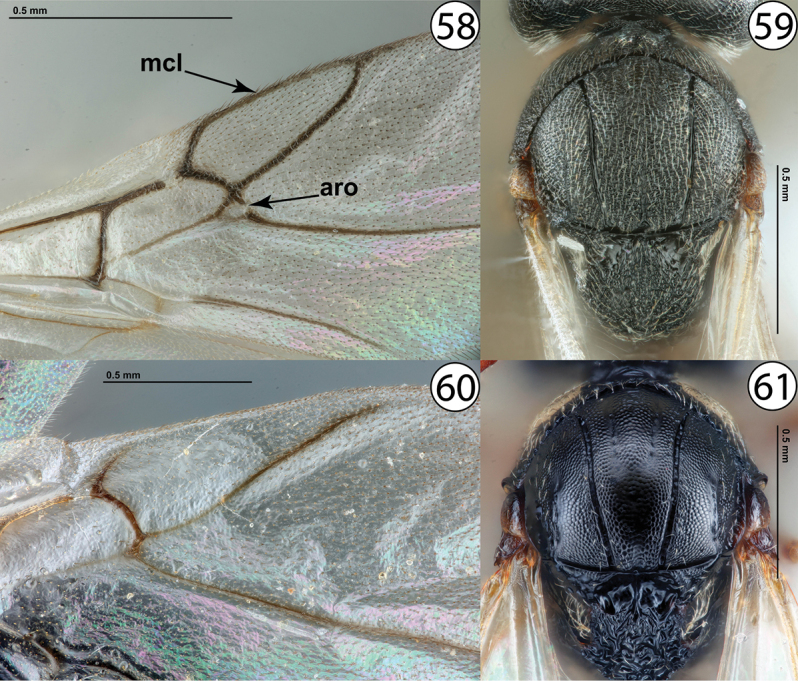
**58***Aulacidea* sp., wings (PSUC_FEM 000247286) **59***Aulacidea* sp., dorsal view (PSUC_FEM 000247286) **60***Liposthenesglechomae*, wings (PSUC_FEM 000248152) **61***Liposthenesglechomae*, dorsal view (PSUC_FEM 000248152). Abbreviations: aro = areolet, mcl = marginal cell.

### ﻿Systematic overview

#### ﻿ Aulacideini

Taxon classificationAnimaliaHymenopteraCynipidae

981653C3-7B17-5876-95D9-8081EAF7484C

Figs 62–67 , 87–89

##### Diagnosis.

Pronotum tall and broad dorsomedially. Pronotal submedial pits distinct and well-impressed. Pronotal plate present, usually only distinct in anterior half of pronotum. Mesopleuron sculpture striate, reticulate, or striate-reticulate. Mesoscutellar foveae distinct. Fore wing with marginal cell entirely open or entirely closed, never partially open. Wings always hyaline, never tinted or with darkened areas. Metatarsal claws without basal lobe. Metasomal tergites 2 and 3 free and articulate, never with a syntergite.

**Figures 62–67.**
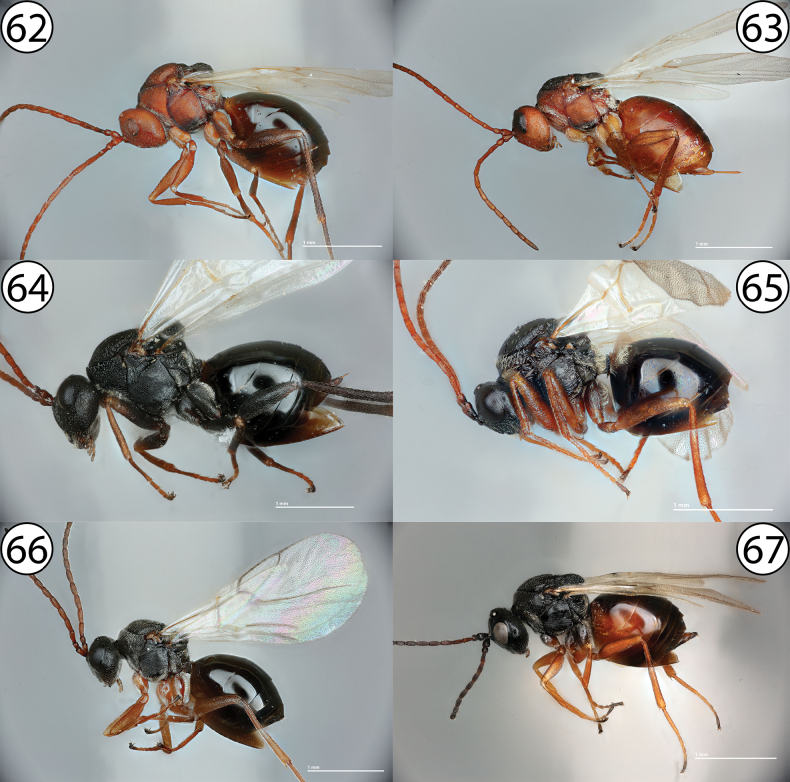
**62***Antistrophuspisum*, lateral view (PSUC_FEM 000247286) **63***Antistrophusmeganae*, lateral view (PSUC_FEM 000248165) **64***Antistrophussilphii*, lateral view (CYNANT0048) **65***Liposthenesglechomae*, lateral view (PSUC_FEM 000248152) **66***Aulacidea* sp., lateral view (PSUC_FEM 000247286) **67***Aulacideahieracii*, lateral view (PSUC_FEM 000253105).

##### Note.

The tribe Aulacideini is represented by approximately 90 species in ten genera worldwide (Nieves-Aldrey 2022), three of which are known from North America (Nastasi and Deans 2021): *Antistrophus* Walsh, 1869, *Aulacidea* Ashmead, 1897, and *Liposthenes* Förster, 1869. Monophyly of the tribe is rather well-established (e.g., Ronquist et al. 2015; Blaimer et al. 2020), but the generic taxonomy is somewhat unsettled (Nieves-Aldrey 2022), and many North American species await description (Nastasi, pers. comm.). The number of introduced described species established in North America is uncertain (see the treatment of *Aulacidea* Ashmead below), but Nastasi and Deans (2021) reported 21 described species.

Globally, members of this tribe induce galls on five plant families (Azmaz and Katılmış 2020; Buffington et al. 2020; Nieves-Aldrey 2022), but the described North American taxa are restricted to host plants in the Asteraceae (tribes Astereae, Chichorieae, and Heliantheae) and Lamiaceae (*Glechomahederacea* L.) (Nastasi and Deans 2021). Galls induced by wasps of this tribe (Figs 87–89) are most likely to yield adults when collected after host plants have senesced; adult wasps emerge in mid spring through late summer depending on the gall wasp species and collecting locality (Nastasi et al., in lit.). Many species induce cryptic galls that produce no externally discernable modification to the plant tissue; this phenomenon suggests that aulacideine herb gall wasps inducing cryptic galls are probably more diverse than currently known and have evaded detection due to their hidden galls.

#### ﻿ Antistrophus

Taxon classificationAnimaliaHymenopteraCynipidae

Walsh, 1869

1D233E5A-47C7-5546-8EB1-15DE735D13A2

##### Type species.

*Antistrophuslygodesmiaepisum* Walsh, 1869 (= *Antistrophuspisum* Ashmead, 1885)

##### Diagnosis.

Mesoscutum sparsely pubescent, at most with scattered setae throughout and never appearing silky. Notauli typically incomplete but complete in several species. Mesopleuron reticulate or striate-reticulate, never entirely transversely striate. Fore wing with marginal cell open, with R1 never reaching anterior wing margin, always without areolet, and with or without marginal setae. Second metasomal tergite without patch of setae.

##### Note.

*Antistrophus* contains ten described species, all of which are known from America north of Mexico (Nastasi and Deans 2021). *Antistrophus* wasps are most commonly encountered in the Eastern and Midwestern United States, although two species, *A.chrysothamni* (Beutenmüller) and *A.microseris* (McCracken & Egbert), are apparently restricted to Arizona and California respectively (Nastasi and Deans 2021). Unpublished records indicate that the genus is far more widely distributed than currently known and is likely common throughout the United States and adjacent parts of Canada (Nastasi, pers. obs.).

Species of *Antistrophus* induce galls on several genera of asteraceous plants: *Chrysothamnus* Nutt.; *Lygodesmia* D.Don; *Microseris* D.Don; and *Silphium* L. Additional plant genera are known to host undescribed species. *Antistrophus* associated with *Silphium* are especially diverse and primarily comprise undescribed species; each *Silphium* species appears to be galled by one or more host-specific or narrowly oligophagous gall wasp species, and some *Antistrophus* are emerging as pests of cultivated *Silphium*.

*Antistrophus*, as currently circumscribed, is a heterogeneous assemblage. The genus contains all North American herb gall wasps that did not fit well within *Aulacidea* Ashmead, 1897 or *Diastrophus* Hartig, 1840, of which the latter is now placed in Diastrophini. Many undescribed species of this genus are known to us, and morphological and molecular data demonstrate that *Antistrophus* as currently defined is poorly circumscribed (unpublished data); the limits of *Antistrophus* will be revised by an ongoing study. Nevertheless, all described species currently placed in this genus as well as all undescribed species currently known to us correctly key to *Antistrophus* here.

###### ﻿North American species (Nastasi and Deans 2021):

1. *Antistrophusbicolor* Gillette, 1891

2. *Antistrophuschrysothamni* (Beutenmüller, 1908)

3. *Antistrophusjeanae* Tooker & Hanks, 2004

4. *Antistrophuslaciniatus* Gillette, 1891

5. *Antistrophusmeganae* Tooker & Hanks, 2004

6. *Antistrophusmicroseris* (McCracken & Egbert, 1922)

7. *Antistrophusminor* Gillette, 1891

8. *Antistrophuspisum* Ashmead, 1885 (replacement name for *A.lygodesmiaepisum* Walsh as given by Nieves-Aldrey [1994] but omitted from Nastasi and Deans [2021])

9. *Antistrophusrufus* Gillette, 1891

10. *Antistrophussilphii* Gillette, 1891

#### ﻿ Aulacidea

Taxon classificationAnimaliaHymenopteraCynipidae

Ashmead, 1897

919C2F42-413C-52CF-9DCB-CA86EB361BF3

##### Type species.

*Aulaxmulgediicola* Ashmead, 1896 (= *Aulacideaharringtoni* [Ashmead, 1897])

##### Diagnosis

(based on North American taxa): Mesoscutum densely pubescent, often appearing silky but at least with rather abundant closely-set setae. Notauli almost always complete (incomplete only in an undescribed species from California). Mesopleuron transversely striate; with a small ventral patch of reticulate sculpture in *Aulacideaacroptilonica* Tyurebaev, 1972. Fore wing with marginal cell entirely closed, with R1 meeting Rs along anterior wing margin, always with areolet, and always with distinct marginal setae. Second metasomal tergite with a distinct patch of setae (absent in *Aulacideaacroptilonica* Tyurebaev, 1972 and sometimes appearing reduced in males of various species).

##### Note.

*Aulacidea* contains some 40 described species (Azmaz and Katılmış 2020; Nieves-Aldrey 2022), 11 of which are known or suspected from North America (Nastasi and Deans 2021). Native species known from North America induce galls primarily on species of *Lactuca* L., although one species (*A.nabali* [Brodie, 1892]) induces galls on *Nabalus* Cass, and one species (*A.ambrosiaecola* [Ashmead, 1896]) is doubtfully associated with *Ambrosia* L. Introduced or suspected species induce galls on *Hieracium* L., *Pilosella* Hill, and *Rhaponticum* Vaill. (Nastasi and Deans 2021).

The number of established exotic *Aulacidea* is problematic as several species have apparently been introduced (e.g., Moffat and Smith 2015), but few records indicate whether they have successfully established. *Aulacideaacroptilonica* Tyurebaev is definitively established in the Pacific Northwest, but it is unclear whether *A.subterminalis* Niblett, 1946 or *A.pilosellae* (Kieffer, 1901) are truly established (Nastasi and Deans 2021). A single *A.pilosellae* was collected via Malaise trap in Canada (Moffat and Smith 2015), but there appear to be no subsequent records indicating establishment of this species in North America. The only accessible evidence of establishment of *A.subterminalis* in North America is a government report detailing introduction attempts in Canada (Government of British Columbia 2018). Records appearing to represent *A.hieracii* (Linnaeus, 1758) on *Hieraciumumbellatum* L. in North America have been confirmed since publication of the recent catalogue, although there are some disputes over whether the population present in the Nearctic is conspecific with those found in the Palearctic (unpublished data). Overall, more research is needed to substantiate the identity and establishment of the introduced taxa.

More generally, *Aulacidea* was erected by Ashmead for herb gall wasps (then, the tribe Aylacini) with a closed marginal cell; this conception of *Aulacidea* remains virtually unchanged at present. As with *Antistrophus*, *Aulacidea* is poorly circumscribed, and the limits of this genus require adjustment (Ronquist et al. 2015; Nieves-Aldrey 2022).

###### ﻿North American species (Nastasi and Deans 2021):

1. *Aulacideaabdita* Kinsey, 1920

2. *Aulacideaacroptilonica* Tyurebaev, 1972

3. *Aulacideaambrosiaecola* (Ashmead, 1896)

4. *Aulacideaannulata* Kinsey, 1920

5. *Aulacideaharringtoni* (Ashmead, 1887)

6. *Aulacideahieracii* (Linnaeus, 1758)

7. *Aulacideanabali* (Brodie, 1892)

8. *Aulacideapilosellae* (Kieffer, 1901)

9. *Aulacideapodagrae* (Bassett, 1890)

10. *Aulacideasubterminalis* Niblett, 1946

11. *Aulacideatumida* (Bassett, 1890)

#### ﻿ Liposthenes

Taxon classificationAnimaliaHymenopteraCynipidae

Förster, 1869

CC4AB371-E9A2-55A5-B2CC-FF02834F6022

##### Type species:

*Aulaxglechomae* Hartig, 1841 (= *Cynipsglechomae* Linnaeus, 1758).

##### Diagnosis.

Mesoscutum sparsely pubescent, at most with a few scattered setae. Notauli complete. Mesopleuron mostly transversely striate, at most with slight indication of reticulate sculpture. Fore wing with marginal cell open, never with areolet distinct, and always with distinct marginal setae. Second metasomal tergite always with a distinct patch of setae.

##### Note.

*Liposthenes* is known in North America from a single introduced species: *L.glechomae* (Linnaeus, 1758). This species was apparently introduced from Europe along with its host plant, *Glechomahederacea* L., and has since become widespread in the United States (Nastasi and Deans 2021). *Liposthenesglechomae* is the only known gall wasp associated with Lamiaceae in the Nearctic; all other known Nearctic Aulacideini, both described and known undescribed species, are associated with Asteraceae.

###### ﻿North American species (Nastasi and Deans 2021):

1. *Liposthenesglechomae* (Linnaeus, 1758)

#### ﻿ Ceroptresini

Taxon classificationAnimaliaHymenopteraCynipidae

B51D64F3-96AF-51E3-BB0E-CA7DD06E166E

Figs 68 , 69

##### Diagnosis.

Pronotum tall and broad dorsomedially. Pronotal submedial pits distinct and well-impressed. Pronotal plate present and complete. Mesoscutellar foveae distinct. Fore wing with marginal cell closed. Metatarsal claws with basal lobe. Metasoma with syntergite, with third tergite greatly enlarged and occupying most of metasoma and with second tergite reduced but free and articulating. First metasomal tergite usually more or less concealed between mesosoma and metasoma and without conspicuous sculpture (more visible and conspicuously striate in some taxa easily confused with *Ceroptres*). Body generally weakly sculptured.

**Figures 68, 69.**
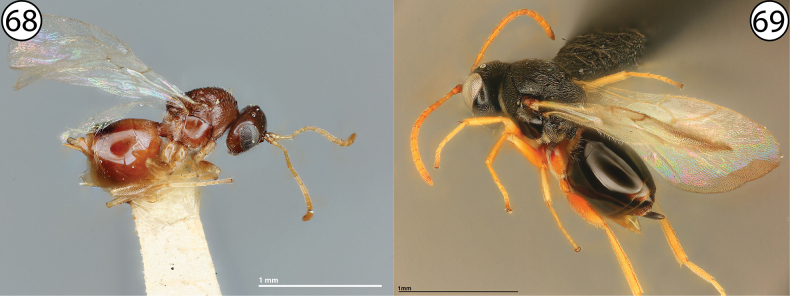
**68***Buffingtonellapolita*, lateral view (USNMENT00892509) **69***Ceroptres* sp., lateral view (USNMENT00917016).

##### Note.

Ceroptresini includes 19 North American species: 18 species of *Ceroptres* Hartig and *Buffingtonellapolita* (Ashmead, 1896) (Nastasi and Deans 2021).

#### ﻿ Buffingtonella

Taxon classificationAnimaliaHymenopteraCynipidae

Lobato-Vila & Pujade-Villar, 2019

267542F4-9CC3-57AD-9176-075CA7EAC182

##### Type species.

*Ceroptrespolitus* Ashmead, 1896

##### Diagnosis.

Area between toruli not depressed and without dense pubescence. Metasomal tergite 1 relatively large and ring-like, not concealed, and longitudinally striate. Frons entirely without facial carinae ventral to toruli.

##### Note.

*Buffingtonella* is known only from Virginia from eight specimens collected in 1884 and 1885 (Lobato-Vila and Pujade-Villar 2019). These specimens were apparently ovipositing into the midribs of leaves of *Quercusrubra* L. at the time of collection, and as such, *B.polita* has been assumed to be an inquiline of an unidentified oak gall wasp (Lobato-Vila and Pujade-Villar 2019). However, the placement of this genus in Ceroptresini, its recognition as distinct from other related taxa, and its biology remain to be substantiated (Lobato-Vila and Pujade-Villar 2019). Upon examining the aforementioned material of this species in the National Museum of Natural History, we confirm the diagnostic characters for the genus as described by Lobato-Vila and Pujade-Villar (2019) and have included it in the above key.

###### ﻿North American species (Nastasi and Deans 2021):

1. *Buffingtonellapolita* (Ashmead, 1896)

#### ﻿ Ceroptres

Taxon classificationAnimaliaHymenopteraCynipidae

Hartig, 1840

A1D832E4-30C7-5443-936E-BC9B124439C6

##### Type species.

*Ceroptresclavicornis* Hartig, 1840.

##### Diagnosis.

Area between toruli distinctly depressed and with abundant pubescence. Metasomal tergite 1 small, mostly concealed between mesosoma and following tergites, and dorsally smooth. Frons with distinct facial carinae ventral to toruli, apparent at least as short ridges (we strongly recommend careful positioning and light diffusion when assessing this character).

##### Note.

*Ceroptres* are occasionally reared from galls induced on oaks by members of the tribe Cynipini (Lobato-Vila and Pujade-Villar 2019; Nastasi and Deans 2021), but are otherwise infrequently encountered. *Ceroptres* are presumed to be inquilines of Cynipini (Ronquist et al. 2015), although some theorize that they may actually be parasitoids due to observation of female *Ceroptres* ovipositing into mature galls rather than developing galls as is typical for gall inquilines (Z. Liu, in lit.). While 18 described species of *Ceroptres* are known from North America, the diversity of this genus has been sparsely surveyed, and many undescribed species are known in association with oak galls (S. Rollins and C. Tribull, pers. comm. 2023). *Ceroptres* have also been reared by several North American research groups in association with galls of cecidomyiid midges, although the exact nature of this association is unknown.

###### ﻿North American species (Nastasi and Deans 2021):

1. *Ceroptrescatesbaei* Ashmead, 1885

2. *Ceroptresconfertus* (McCracken & Egbert, 1922)

3. *Ceroptrescornigera* Melika & Buss, 2002

4. *Ceroptresensiger* (Walsh, 1864)

5. *Ceroptresfrondosae* Ashmead, 1896

6. *Ceroptresjunquerasi* Lobato-Vila & Pujade-Villar, 2019

7. *Ceroptreslanigerae* Ashmead, 1885

8. *Ceroptreslenis* Lobato-Vila & Pujade-Villar, 2019

9. *Ceroptresmexicanus* Lobato-Vila & Pujade-Villar, 2019

10. *Ceroptresminutissimi* Ashmead, 1885

11. *Ceroptresmontensis* Weld, 1957

12. *Ceroptresnigricus* Lobato-Vila & Pujade-Villar, 2019

13. *Ceroptrespetiolicola* (Osten Sacken, 1861)

14. *Ceroptrespisum* (Osten Sacken, 1861)

15. *Ceroptresquadratifacies* Lobato-Vila & Pujade-Villar, 2019

16. *Ceroptresrufiventris* Ashmead, 1896

17. *Ceroptressnellingi* Lyon, 1996

#### ﻿ Cynipini

Taxon classificationAnimaliaHymenopteraCynipidae

30539C33-374A-572D-8699-018B414DE164

Figs 70–72

##### Diagnosis.

Pronotum distinctly short and narrow dorsomedially, without distinct plate or pits. Scutellar foveae usually distinct. Mesopleuron usually without broad crenulate impression. Female hypopygium only very rarely plowshare-shaped; only so in *Protobalandricus* Melika, Nicholls & Stone, 2018, in which the mesopleuron is entirely smooth and therein readily separable from *Diplolepis* Geoffroy, 1762 (Cuesta Porta, pers. comm. 13 Feb 2024).

**Figures 70–72.**
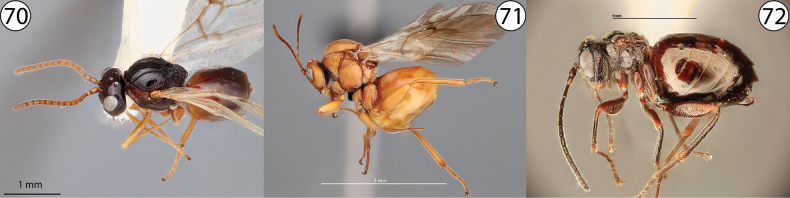
**70***Dryocosmuskuriphilus*, lateral view (USNMENT01231861) **71***Andricusquercuscalifornicus*, lateral view (USNMENT01231839) **72***Phylloteras* sp., lateral view (USNMENT01231835).

##### Note.

Cynipini is represented by an estimated 680 North American species that induce galls primarily on *Quercus* (Fagaceae) (Melika et al. 2021). Additional host genera known are *Castanea* Mill., *Chrysolepis* Hjelmq., and *Notholithocarpus* Manos, Cannon, & S.H. Oh (Buffington and Morita 2009). Genera belonging to Cynipini are not keyed in the present work due to the presence of several highly unstable genera that prohibit clear morphological recognition, although recent studies (e.g., Melika et al. 2021) have made taxonomic changes that greatly ease this burden. Further revisionary studies will continue to stabilize genera in the Cynipini, and a key to Cynipini will be published when possible. Relevant keys for Cynipini include Weld (1952), Zimmerman (2018), and Melika et al. (2021), but these works are partial in their taxon coverage or do not align well with current taxonomic hypotheses.

#### ﻿ Diastrophini

Taxon classificationAnimaliaHymenopteraCynipidae

2D1172A4-CEE2-5D68-9936-93A993A40C34

Figs 73–75 , 92–94

##### Diagnosis.

Pronotum tall and broad dorsomedially. Pronotal submedial pits distinct and well-impressed. Pronotal plate present and complete, distinct both dorsally and ventrally. Mesopleuron sculpture striate or smooth and shining. Mesoscutellar foveae distinct. Fore wing with marginal cell entirely open or entirely closed, never partially open. Wings often with darkened areas, especially around the marginal cell. Metatarsal claws always with basal lobe. Metasomal tergites 2 and 3 either free and articulate, or fused into a syntergite in some females.

**Figures 73–75.**
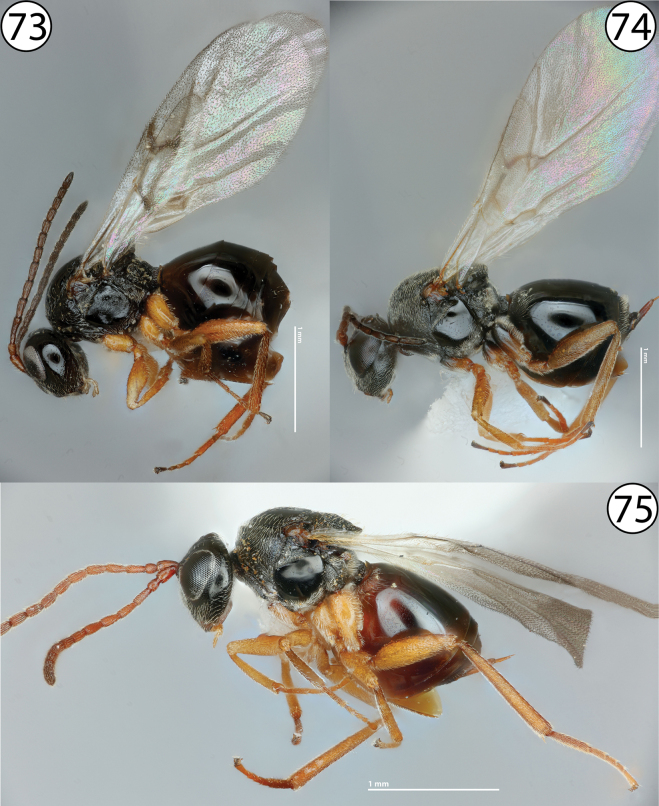
**73***Diastrophuskincaidii*, lateral view (PSUC_FEM 000251280) **74***Periclistus* sp., lateral view (PSUC_FEM 000250920) **75***Synophromorpha* sp., lateral view (PSUC_FEM 000250918).

##### Note.

Diastrophini includes 25 described North American species in three genera: *Diastrophus* Hartig, 1840, *Periclistus* Förster, 1869, and *Synophromorpha* Ashmead, 1903 (Nastasi and Deans 2021). The North American members of this tribe are gall inducers on various Rosaceae or inquilines in the galls of *Diastrophus* Hartig, 1840 or *Diplolepis* Geoffroy, 1762 (Nastasi and Deans 2021).

#### ﻿ Diastrophus

Taxon classificationAnimaliaHymenopteraCynipidae

Hartig, 1840

C9FBDBE8-B9F8-5ECC-8F79-1F7A80FC9960

##### Type species.

*Cynipsrubi* Bouché, 1834.

##### Diagnosis.

Mesoscutum generally weakly sculptured and without abundant strong setigenous punctures. Notauli complete and strong throughout. Mesopleuron sculpture smooth to striate. Fore wing with marginal cell open. Metasoma never with syntergite.

##### Note.

*Diastrophus* contains 14 North American species (Nastasi and Deans 2021). Many species induce galls on *Rubus* L., although the herbaceous genera *Fragaria* L. and *Potentilla* L. are also used. *Diastrophussmilacis* Ashmead, 1896 and its supposed inquiline, *Periclistussmilacis* Ashmead, 1896, were previously believed to be associated with *Smilax* L., making *D.smilacis* the only cynipid known to induce galls on a monocot plant (Gates et al. 2020). However, Gates et al. conclude that this association was erroneous, and the true gall inducer on *Smilax* is in fact a eulophid wasp (Chalcidoidea: *Aprostocetussmilax* Gates & Zhang). The biological associations of Diastrophini therein are still atypical as *Periclistus* inquilines are generally associated with the tribe Diplolepidini. Our own examination of the type material of *D.smilacis* and *P.smilacis* (deposited in the USNM) confirm that they are indeed placed in the appropriate genera, although the status of either species and their biological relationships remain suspect and require further investigation.

Galls of Diastrophini (Figs 92–94) can be collected for rearing in the fall, winter, or spring. As in Aulacideini, galls on herbaceous hosts are best collected after host plants have senesced, and adults of all *Diastrophus* emerge in spring and summer.

###### ﻿North American species (Nastasi and Deans 2021):

1. *Diastrophusaustrior* Kinsey, 1922

2. *Diastrophusbassettii* Beutenmüller, 1892

3. *Diastrophuscuscutaeformis* Osten Sacken, 1863

4. *Diastrophusfragariae* Beutenmüller, 1915

5. *Diastrophusfusiformans* Ashmead, 1890

6. *Diastrophuskincaidii* Gillette, 1893

7. *Diastrophusnebulosus* (Osten Sacken, 1861)

8. *Diastrophusniger* Bassett, 1900

9. *Diastrophuspiceus* Provancher, 1886

10. *Diastrophuspotentillae* Bassett, 1864

11. *Diastrophusradicum* Bassett, 1870

12. *Diastrophussmilacis* Ashmead, 1896

13. *Diastrophustumefactus* Kinsey, 1920

14. *Diastrophusturgidus* Bassett, 1870

#### ﻿ Periclistus

Taxon classificationAnimaliaHymenopteraCynipidae

Förster, 1869

EA834995-DD1E-5E18-8785-CC99D93EB1D7

##### Type species.

*Aylaxcaninae* Hartig, 1840.

##### Diagnosis.

Mesoscutum generally coarsely sculptured, usually densely pubescent, and with abundant strong setigenous punctures. Notauli incomplete, indistinct at least in anterior third, and weaker throughout. Fore wing with marginal cell closed. Metasoma with syntergite in females but with all tergites free and articulating in males.

##### Note.

*Periclistus* contains seven North American species, all of which are inquilines of *Diplolepis* Geoffroy inducing galls on species of *Rosa* L., except for *P.smilacis* Ashmead (see treatment of *Diastrophus* Hartig). The diversity of this genus is not well understood; Ritchie (1984) treated ten Nearctic species in his unpublished thesis including six new species, but a recent DNA barcoding study (Zhang et al. 2019) revealed the presence of two undescribed Nearctic species. More broadly, future study is needed to investigate host associations, especially given the presence of undescribed species.

###### ﻿North American species (Nastasi and Deans 2021):

1. *Periclistusarefactus* McCracken & Egbert, 1922

2. *Periclistuscalifornicus* Ashmead, 1896

3. *Periclistusobliquus* Provancher, 1888

4. *Periclistuspiceus* Fullaway, 1911

5. *Periclistuspirata* (Osten Sacken, 1863)

6. *Periclistussemipiceus* (Harris, 1841)

7. *Periclistussmilacis* Ashmead, 1896

#### ﻿ Synophromorpha

Taxon classificationAnimaliaHymenopteraCynipidae

Ashmead, 1903

FB4B49D3-90DB-52A4-B693-5E88504E1CEE

##### Type species.

*Synophrussylvestris* Osten Sacken, 1861.

##### Diagnosis.

Mesoscutum generally less coarsely sculptured, appearing mostly or entirely coriaceous, less pubescent, and with some strong setigenous punctures. Notauli complete, strong throughout. Fore wing with marginal cell open. Metasoma with syntergite in females but with all tergites free and articulating in males.

##### Note.

*Synophromorpha* is represented by four species in North America, all of which are inquilines of *Diastrophus* species associated with *Rubus* L. Ritchie and Shorthouse (1987) described the species *S.kaulbarsi* Shorthouse & Ritchie, 1987 from a single specimen collected in Mexico; they speculated that this species was evidence of undiscovered Mexican *Diastrophus* or represented the use of an alternative host such as an oak gall wasp.

###### ﻿North American species (Nastasi and Deans 2021):

1. *Synophromorphakaulbarsi* Ritchie & Shorthouse, 1987

2. *Synophromorpharubi* Weld, 1952

3. *Synophromorphasylvestris* (Osten Sacken, 1861)

4. *Synophromorphaterricola* Weld, 1952

#### ﻿ Diplolepis

Taxon classificationAnimaliaHymenopteraCynipidae

Geoffroy, 1762 (Diplolepididae: Diplolepidinae)

1B6BF731-560C-53EB-883F-D7ADBB6E8E1E

**Figures 76–79.**
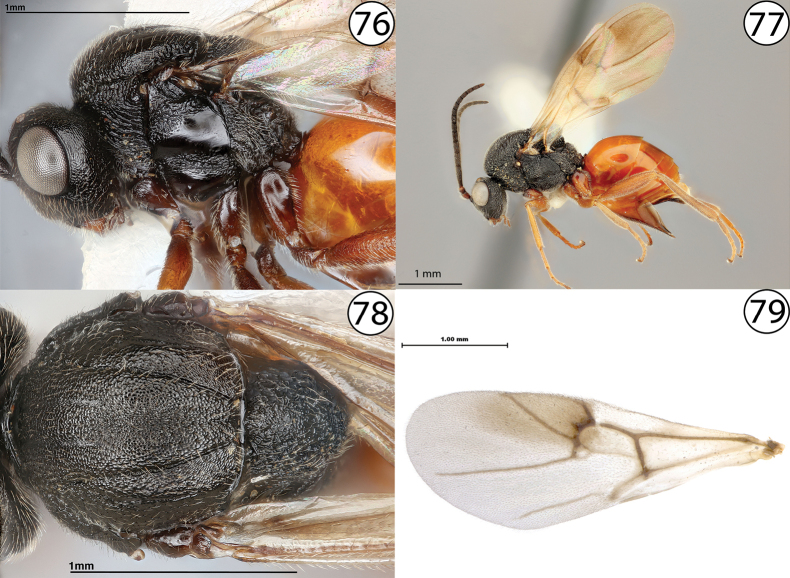
**76***Diplolepisbicolor*, lateral view (USNMENT01231831) **77***Diplolepisbicolor*, lateral view (USNMENT01231831) **78***Diplolepisbicolor*, dorsal view (USNMENT01231831) **79***Diplolepisrosae*, fore wing (USNMENT00655959).

Figs 76–79 , 90 , 91

##### Type species.

*Cynipsrosae* Linnaeus, 1758.

##### Diagnosis.

Pronotum distinctly short and narrow dorsomedially, without distinct plate or pits. Scutellar foveae faint or absent, never distinct and well impressed. Mesopleuron with broad crenulate medial impression. Female hypopygium plowshare-shaped.

##### Note.

Diplolepidinae includes 34 described North American species in *Diplolepis* Geoffroy which induce structurally diverse galls (Figs 90, 91) on *Rosa* (Rosaceae) and are widely distributed in the US and Canada (Nastasi and Deans 2021). Recent phylogenomic studies (Blaimer et al. 2020; Hearn et al. 2023) showed that the tribe Diplolepidini clustered together with Pediaspidini outside of the core Cynipidae, causing Cynipidae to form a paraphyletic grade at the base of Cynipoidea. Hearn et al. (2023) raised the former tribe Diplolepidini to subfamily rank (Diplolepidinae) within the family Diplolepididae. All other taxa treated here remain in the Cynipidae.

###### ﻿North American species (Nastasi and Deans 2021):

1. *Diplolepisarefacta* (Gillette, 1894)

2. *Diplolepisashmeadi* (Beutenmüller, 1918)

3. *Diplolepisbassetti* (Beutenmüller, 1918)

4. *Diplolepisbicolor* (Harris, 1841)

5. *Diplolepiscalifornica* (Beutenmüller, 1914)

6. *Diplolepisdichlocera* (Harris, 1841)

7. *Diplolepisfulgens* (Gillette, 1894)

8. *Diplolepisfusiformans* (Ashmead, 1890)

9. *Diplolepisgracilis* (Ashmead, 1897)

10. *Diplolepisignota* (Osten Sacken, 1863)

11. *Diplolepisinconspicuis* Dailey & Campbell, 1973

12. *Diplolepislens* Weld, 1952

13. *Diplolepismayri* (Schlechtendal, 1877)

14. *Diplolepisnebulosa* (Bassett, 1890)

15. *Diplolepisneglecta* (Gillette, 1894)

16. *Diplolepisnervosa* (Curtis, 1838)

17. *Diplolepisnodulosa* (Beutenmüller, 1909)

18. *Diplolepisoregonensis* (Beutenmüller, 1918)

19. *Diplolepisostensackeni* (Beutenmüller, 1918)

20. *Diplolepispolita* (Ashmead, 1890)

21. *Diplolepispustulatoides* (Beutenmüller, 1914)

22. *Diplolepisradicum* (Osten Sacken, 1863)

23. *Diplolepisrosae* (Linnaeus, 1758)

24. *Diplolepisrosaefolii* (Cockerell, 1889)

25. *Diplolepissimilis* (Ashmead, 1896)

26. *Diplolepisspinosa* (Ashmead, 1887)

27. *Diplolepisterrigena* Weld, 1952

28. *Diplolepistriforma* Shorthouse & Ritchie, 1984

29. *Diplolepistuberculator* (Cockerell, 1888)

30. *Diplolepistuberculosa* (Osten Sacken, 1861)

31. *Diplolepistumida* (Bassett, 1890)

32. *Diplolepisvariabilis* (Bassett, 1890)

33. *Diplolepisverna* (Osten Sacken, 1863)

34. *Diplolepisweldi* (Beutenmüller, 1913)

#### ﻿ Phanacis

Taxon classificationAnimaliaHymenopteraCynipidae

Förster, 1860 (Phanacidini)

EBC146B8-4F12-5ED5-B850-57169C26BD71

Figs 80–82 , 95

##### Type species.

*Parapanteliellaeugeniae* Diakontschuk, 1981.

##### Diagnosis.

Pronotum tall and broad dorsomedially. Pronotal submedial pits rather indistinct and poorly impressed, appearing as a narrow linear impression rather than distinct ovular pits. Pronotal plate present, usually only distinct in anterior half of pronotum. Mesopleuron sculpture reticulate. Mesoscutellar foveae distinct. Fore wing with marginal cell partially open, with vein R1 reaching anterior margin of fore wing and continuing along wing margin but not meeting vein Rs. Wings always hyaline, never tinted or with darkened areas. Metatarsal claws without basal lobe. Metasomal tergites 2 and 3 free and articulate, never with a syntergite.

**Figures 80–82.**
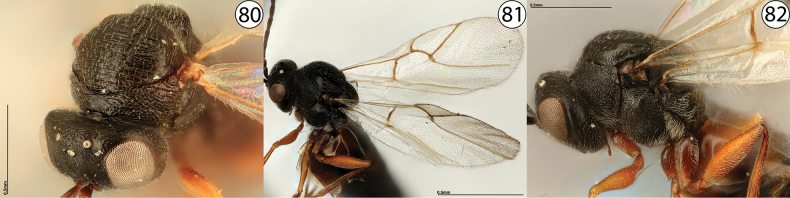
**80***Phanacis* sp., anterodorsal view (USNMENT01448498) **81***Phanacis* sp., wings (USNMENT01231855) **82***Phanacis* sp., lateral view (USNMENT01231855).

##### Note.

Phanacidini includes two North American species, both in *Phanacis* Förster, which have been introduced along with their host plants (Nastasi and Deans 2021). *Phanacishypochoeridis* (Kieffer, 1887) induces galls on *Hypochaerisradicata* L. and is apparently restricted to the western United States (Nastasi and Deans 2021). The other species, *P.taraxaci* (Ashmead, 1897), induces galls on *Taraxacumofficinale* F. H. Wigg. (Fig. 95) and is widespread in eastern North America (Nastasi and Deans 2021).

###### ﻿North American species (Nastasi and Deans 2021):

1. *Phanacishypochoeridis* (Kieffer, 1887)

2. *Phanacistaraxaci* (Ashmead, 1897)

#### ﻿ Synergus

Taxon classificationAnimaliaHymenopteraCynipidae

Hartig, 1840 (Synergini)

F3875393-5486-52E8-A5E0-41E7FD45E6D4

Figs 83–86

##### Type species.

*Synergusvulgaris* Hartig, 1840.

##### Diagnosis.

Pronotum tall and broad dorsomedially. Pronotal submedial pits distinct and well-impressed. Pronotal plate present but mostly indistinct. Mesoscutellar foveae usually distinct. Fore wing always with marginal cell closed (apparently only partly closed in *Synergusmexicanus* Gillette, 1896; see Pujade-Villar et al. 2015). Metatarsal claws with or without basal lobe. Metasoma with syntergite, with second and third tergites entirely fused, greatly enlarged, and occupying most of metasoma. Body generally strongly sculptured.

**Figures 83–86.**
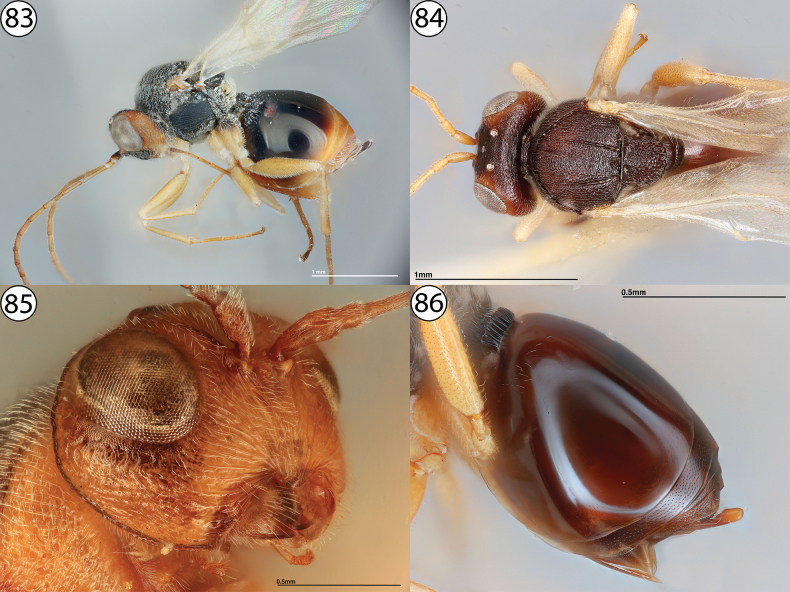
**83***Synergus* sp., lateral view (PSUC_FEM 000079457) **84***Synergusincisus*, dorsal view (USNMENT01231859) **85***Synerguslignicola*, anterior view (USNMENT01448497) **86***Synergus* sp., metasoma, lateral view (USNMENT01231858).

##### Note.

Sixty-one species of *Synergus* are known from North America (Nastasi and Deans 2021). Members of *Synergus* Hartig are inquilines of galls induced by Cynipini on oaks (Buffington et al. 2020). *Synergus* are extremely commonly reared and are known in association with hundreds of oak gall wasps (Nastasi and Deans 2021; Ward et al. 2022). *Synergus* is demonstrably polyphyletic, with North American taxa forming as many as three independent clades and many undescribed species exist (Pénzes et al. 2012; Lobato-Vila and Pujade-Villar 2021; Lobato-Vila et al. 2022), meaning a great deal of revisionary work will be needed to resolve major questions within the genus and better understand its diversity.

**Figures 87–95.**
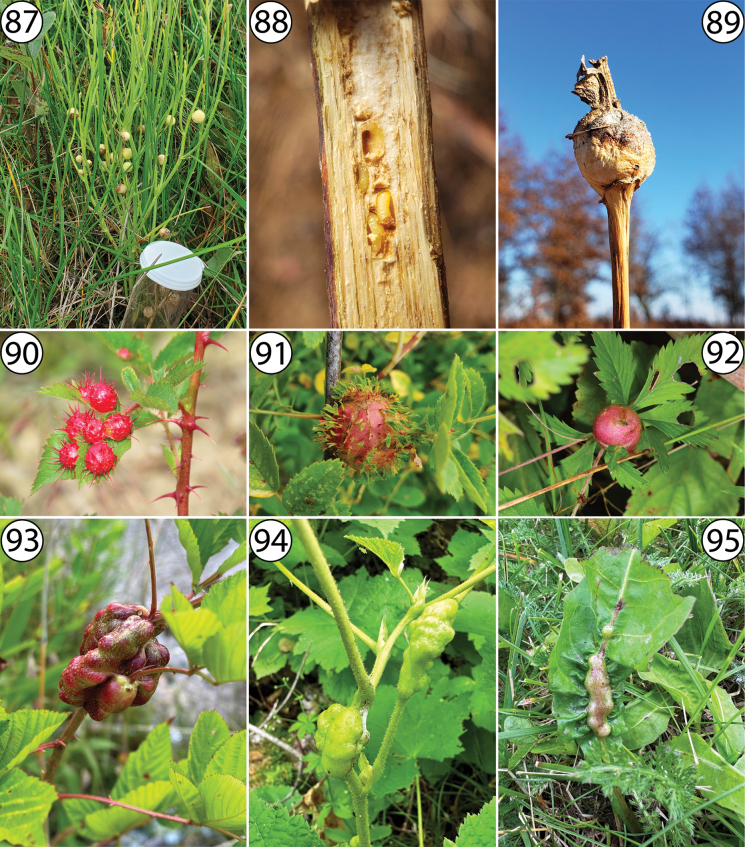
**87** galls of *Antistrophuspisum* on stem of *Lygodesmiajuncea* (Asteraceae: Cichorieae), photographed by Chris Friesen (https://www.inaturalist.org/observations/95588437) **88** galls of *Antistrophusrufus* in dissected stem of *Silphiumlaciniatum* (Asteraceae: Heliantheae), photographed by Andy Deans (https://www.inaturalist.org/observations/64708490) **89** gall of *Antistrophussilphii* on apical stem of *Silphiumintegrifolium* (Asteraceae: Heliantheae), photographed by Andy Deans (https://www.inaturalist.org/observations/64708191) **90** galls of *Diplolepispolita* on leaves of *Rosa* sp. (Rosaceae: Roseae), photographed by Garth Harwood (https://www.inaturalist.org/observations/165442438) **91** gall of *Diplolepiscalifornica* on *Rosa* sp. (Rosaceae: Roseae), photographed by Mary K. Hanson (https://www.inaturalist.org/observations/115655737) **92** gall of *Diastrophuspotentillae* on *Potentillasimplex* (Rosaceae: Potentilleae), photographed by Tom Murray (https://www.inaturalist.org/observations/134669544) **93** gall of *Diastrophusnebulosus* on stem of *Rubus* sp. (Rosaceae: Rubeae), photographed by Pam Curtin (https://www.inaturalist.org/observations/174007397) **94** galls of *Diastrophuskincaidii* on stems of *Rubusparviflorus* (Rosaceae: Rubeae), photographed by Adam Heikkila (https://www.inaturalist.org/observations/173314109) **95** galls of *Phanacistaraxaci* on petiole of *Taraxacumofficinale* (Asteraceae: Cichorieae), photographed by Nathan Earley (https://www.inaturalist.org/observations/174118397).

The genus *Saphonecrus* Dalla Torre & Kieffer (Tribe Synergini) has long been considered present in North America, but recent taxonomic work refutes this idea. Nastasi and Deans (2021) reported two species: *S.favanus* Weld and *S.gemmariae* (Ashmead). *Saphonecrusgemmariae* was reported in error as the species was considered incertae sedis by Lobato-Vila et al. (2022) due to missing type material which was supposedly deposited in the National Museum of Natural History (USA, D.C.). Upon our own examination of the USNM collection, we were unable to locate the relevant type material. Similarly, the status of *S.favanus* is also questionable (Pénzes et al. 2009; Pénzes et al. 2012; Lobato-Vila and Pujade-Villar 2021); this species may represent a new genus distinct from other Synergini (Lobato-Vila et al. 2022). As such, we consider the presence of *Saphonecrus* in North America doubtful and have omitted *Saphonecrus* from the above key. We have examined type material of *S.favanus* deposited in USNM (specimen # USNMENT960420 and three additional individuals) and found that in the key to genera, the specimens key to *Synergus*, bearing no strong distinction from this genus. The taxonomy of the tribe Synergini as a whole is currently uncertain, and ongoing efforts to revise it will likely result in a stronger understanding of the North American fauna (Lobato-Vila et al. 2022).

###### ﻿North American species (Nastasi and Deans 2021):

1. *Synergusagrifoliae* Ashmead, 1896

2. *Synergusashmeadi* Lobato-Vila & Pujade-Villar, 2021

3. *Synergusaurofacies* Lobato-Vila & Pujade-Villar, 2020

4. *Synergusatra* Gillette, 1896

5. *Synergusatripennis* Ashmead, 1896

6. *Synergusatripes* Gillette, 1896

7. *Synergusbatatoides* Ashmead, 1885

8. *Synergusbellus* McCracken & Egbert, 1922

9. *Synergusbeutenmulleri* Lobato-Vila & Pujade-Villar, 2021

10. *Synergusbrevicornis* Ashmead, 1896

11. *Synergusbicolor* Ashmead, 1885

12. *Synerguscampanula* Osten Sacken, 1865

13. *Synerguscastanopsidis* (Beutenmüller, 1918)

14. *Synerguscibriani* Lobato-Vila & Pujade-Villar, 2017

15. *Synerguscitriformis* (Ashmead, 1885)

16. *Synerguscompressus* Lobato-Vila & Pujade-Villar, 2021

17. *Synergusconfertus* McCracken & Egbert, 1922

18. *Synergusconiferae* Ashmead, 1885

19. *Synergusdigressus* McCracken & Egbert, 1922

20. *Synergusdimorphus* Osten Sacken, 1865

21. *Synergusdistinctus* McCracken & Egbert, 1922

22. *Synergusdiversicolor* Lobato-Vila & Pujade-Villar, 2021

23. *Synergusdorsalis* (Provancher, 1888)

24. *Synergusduricorius* Gillette, 1896

25. *Synergusebenus* Lobato-Vila & Pujade-Villar, 2021

26. *Synergusequihuai* Pujade-Villar & Lobato-Vila, 2016

27. *Synerguserinacei* Gillette, 1896

28. *Synergusestradae* Pujade-Villar & Lobato-Vila, 2016

29. *Synergusficigerae* Ashmead, 1885

30. *Synergusfilicornis* Cameron, 1883

31. *Synergusflavens* McCracken & Egbert, 1922

32. *Synergusforcadellae* Lobato-Vila & Pujade-Villar, 2020

33. *Synergusgilletti* Pujade-Villar & Lobato-Vila, 2017

34. *Synergusgrahami* Lobato-Vila & Pujade-Villar, 2019

35. *Synergusincisus* Gillette, 1896

36. *Synerguslaeviventris* (Osten Sacken, 1861)

37. *Synerguslignicola* (Osten Sacken, 1862)

38. *Synerguslinnei* Lobato-Vila & Pujade-Villar, 2021

39. *Synerguslongimalaris* Pujade-Villar & Lobato-Vila, 2017

40. *Synerguslongiscapus* Pujade-Villar & Lobato-Vila, 2017

41. *Synergusmacrackenae* Lobato-Vila & Pujade-Villar, 2021

42. *Synergusmedullae* Ashmead, 1885

43. *Synergusmendax* Walsh, 1864

44. *Synergusmexicanus* Gillette, 1896

45. *Synergusnigroornatus* McCracken & Egbert, 1922

46. *Synergusoaxaquensis* Lobato-Vila & Pujade-Villar, 2021

47. *Synergusobtusilobae* (Ashmead, 1885)

48. *Synergusochreus* Fullaway, 1911

49. *Synergusoneratus* (Harris, 1841)

50. *Synerguspacificus* McCracken & Egbert, 1922

51. *Synerguspersonatus* Lobato-Vila & Pujade-Villar, 2021

52. *Synerguspomiformis* Ashmead, 1885

53. *Synerguspseudofilicornis* Lobato-Vila & Pujade-Villar, 2018

54. *Synerguspunctatus* Gillette, 1896

55. *Synergusquercuslana* (Fitch, 1859)

56. *Synergusreniformis* McCracken & Egbert, 1922

57. *Synergusruficephalus* Lobato-Vila & Pujade-Villar, 2021

58. *Synergusrutulus* McCracken & Egbert, 1922

59. *Synergusshorthousei* Lobato-Vila & Pujade-Villar, 2019

60. *Synergusstelluli* Burnett, 1976

61. *Synergusstratifrons* Pujade-Villar & Lobato-Vila, 2017

62. *Synergussuccinipedis* (Ashmead, 1885)

63. *Synergustenebrosus* Lobato-Vila & Pujade-Villar, 2019

64. *Synergusvillosus* Gillette, 1891

65. *Synergusvirentis* (Ashmead, 1885)

66. *Synerguswalshii* Gillette, 1896

67. *Synergusweldi* Lobato-Vila & Pujade-Villar, 2021

## Supplementary Material

XML Treatment for Aulacideini

XML Treatment for Antistrophus

XML Treatment for Aulacidea

XML Treatment for Liposthenes

XML Treatment for Ceroptresini

XML Treatment for Buffingtonella

XML Treatment for Ceroptres

XML Treatment for Cynipini

XML Treatment for Diastrophini

XML Treatment for Diastrophus

XML Treatment for Periclistus

XML Treatment for Synophromorpha

XML Treatment for Diplolepis

XML Treatment for Phanacis

XML Treatment for Synergus

## ﻿Additional information

### Author ORCIDs

Louis F. Nastasi https://orcid.org/0000-0001-7825-480X

Matthew L. Buffington https://orcid.org/0000-0003-1900-3861

Charles K. Davis https://orcid.org/0000-0001-6056-3903

Andrew R. Deans https://orcid.org/0000-0002-2119-4663

### Data availability

All of the data that support the findings of this study are available in the main text.
